# Investigation on photopolymerization of PEGDA to fabricate high-aspect-ratio microneedles†

**DOI:** 10.1039/d2ra00189f

**Published:** 2022-03-28

**Authors:** Sohyun Kim, Hyemin Lee, Hyewon Choi, Kee-Youn Yoo, Hyunsik Yoon

**Affiliations:** Department of Nano Bio Engineering, Seoul National University of Science and Technology Seoul 01811 Republic of Korea hsyoon@seoultech.ac.kr; Department of Chemical & Biomolecular Engineering, Seoul National University of Science and Technology Seoul 01811 Republic of Korea kyyoo@seoultech.ac.kr

## Abstract

Microneedles (MNs) are micron-sized needles that can penetrate the stratum corneum, enabling the non-invasive and painless administration of drugs and vaccines. In this work, fabrication conditions for high-aspect-ratio MNs by the photopolymerization of polyethylene glycol diacrylate (PEGDA) were investigated. Ultraviolet (UV) light was used to crosslink photocurable prepolymers in specific areas defined by a photomask. The aspect ratio of solidified MNs is too small to penetrate the stratum corneum if the degree of polymerization is insufficient. However, if the degree of polymerization is too high, a film is formed between the MNs by solidification of an undesired area owing to the scattering effect, reducing needle height. The influence of prepolymer molecular weight and the degree of UV absorption by the photoinitiator (PI) were studied to optimize the conditions for obtaining high-aspect-ratio MNs. Additionally, the effect of spacing ratio on high-aspect-ratio MNs without film formation has been discussed. A penetration test was conducted with porcine skin to analyze the effect of mechanical properties of MN. This study could guide the fabrication of MNs by the photopolymerization of biocompatible polymers with a photomask.

## Abstract 2

Fabrication conditions for high-aspect-ratio MNs by the photopolymerization of PEGDA were investigated. The influence of prepolymer molecular weight and the degree of UV absorption by the photoinitiator (PI) were studied to optimize the conditions.

## Graphical abstract

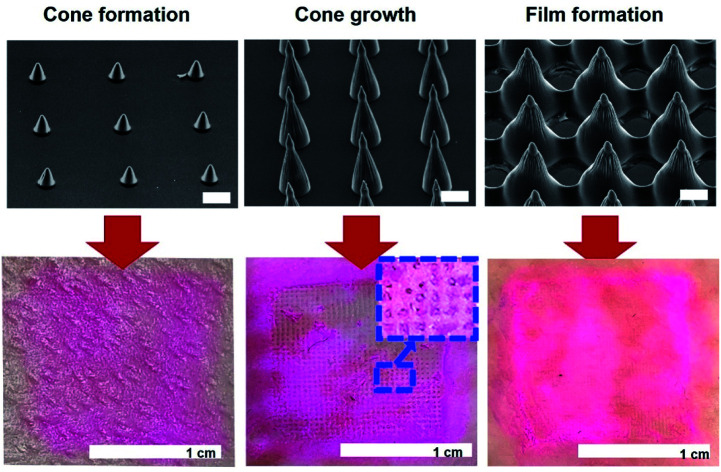

## Introduction

Transdermal drug delivery has attracted immense attention in the pharmaceutical field, as it is a good alternative to the oral delivery system and traditional hypodermic injections.^1^ Through transdermal drug delivery, the drug penetrates the stratum corneum, which is the physical barrier of the epidermis, and is absorbed into the dermal layer. It bypasses the first-pass effect of the liver, which reduces the dose before reaching the target.^2^ However, only a few drugs can be transdermal drug delivery candidates, because of their resistance to drug transport by the stratum corneum.^3^ This limitation could be overcome by micron-sized needles, which allow drugs to pass through the stratum corneum.

Microneedles (MNs) enabling non-invasive and painless administration of drugs are one of the most promising transdermal drug delivery systems.^4^ MNs can be made of metal, silicon, and polymers.^5^ Metal MNs, although mechanically strong and biocompatible, are dangerous because of the possibility of breakage underneath the skin. Microfabrication devices, such as micro-mechanical–electrical systems (MEMS), have used silicon as base material, suggesting its use for MN fabrication.^6^ Although the processing of silicon is well established, its complexity of fabrication could be a bottleneck for widespread use. Polymers, such as polylactic acid (PLA), polylactic-co-glycolic acid (PLGA), and polyethylene glycol (PEG), which are biocompatible, could be used to fabricate MNs.^7–9^ Generally, a molding method is used for polymer fabrication.^10^ A mold is prepared using laser ablation, or microfabrication (such as photolithography), followed by dry etching. Subsequently, solidified polymers are obtained by filling polymers into the mold void. A mold with the shape of a hole array generates a polymer having the shape of an MN array. This method is useful for molding various kinds of polymers; however, it is difficult to fabricate complicated three-dimensional structures.^11^ Another fabrication method for polymers is the direct photopolymerization of prepolymers using a photomask.^12–16^ For monolithic photopolymerization, photocurable prepolymers mixed with photoinitiators (PI) are generally used. Prepolymers have ultraviolet (UV)-curable functional groups, such as acrylates, with double bonds that react with free radicals. After the UV exposure, PIs generate free radicals to polymerize prepolymers with acrylate groups.^13^ The degree of polymerization is controlled by varying the exposure time and PI concentration. A photomask induces selective photopolymerization with the transparent region of the mask transmitting a UV light. For instance, snake-fang-inspired MNs with multiple grooves can be realized by designing a photomask with multiple blade patterns.^12^ The groove can enhance the drug filling through MN surfaces owing to the capillary action. Another example is the fabrication of symmetric and asymmetric hollow MNs by one-shot photopolymerization with a designed photomask.^14^ By designing the photomask, MNs with particular shapes can be fabricated, which is an advantage of the direct photopolymerization.^12–16^ Many geometrical factors such as shape, tip diameter, height, force and velocity of insertion, and density of MN arrays and materials, should be considered before fabricating MNs.^17^ MN height is a particularly important geometric parameter that affects the possibility of stratum corneum penetration. Although there are several reports on the fabrication of MNs by photopolymerization, very few studies have investigated the effect of parameters, such as prepolymer molecular weight, and PI absorption, on the geometry of fabricated MNs. If the exposure time is too short, and the molecular weight of the prepolymer is low, the length of the MN is too short to penetrate the stratum corneum. If the exposure time is too long and the molecular weight of the prepolymer is too high, the polymerization region spreads in the horizontal direction, forming a film between the MNs, generating an effect that shortens the height of the MNs. In this study, conditions for fabricating high-aspect-ratio PEGDA were investigated. Polyethylene glycol diacrylate (PEGDA) is one of the widely used biocompatible hydrogel materials approved by the Food and Drug Administration (FDA).^18,19^ It can be used as a photocurable prepolymer for the fabrication of MNs by photopolymerization with a photomask.^19^ In this study, we investigated the condition for realizing high-aspect-ratio MNs by a photopolymerization to penetrate the stratum conium. Effects of the prepolymer molecular weight, and PI absorption coefficients on the fabrication of high-aspect-ratio MNs, were analyzed.

Additionally, the design parameter, and the spacing ratio between MNs, to attain optimum conditions for MN penetration into the stratum corneum were studied. After MN fabrication on a flexible film, a penetration test was conducted on porcine skin.

## Experimental

### Materials

Hydrogel polyethylene glycol diacrylate (PEGDA, molecular weight: 250, 575, 700) and 2-hydroxy-2-methylpropiophenone (Darocur 1173), 1-hydroxycyclohexyl phenyl ketone (Irgacure 184), and 2,2-dimethoxy-2-phenyl acetophenone (Irgacure 651) were purchased from Sigma-Aldrich, USA. PEGDA was mixed with 5 wt% of PI as a prepolymer. Polydimethylsiloxane (PDMS) was purchased from Dow Corning.

### Photolithography process to fabricate MNs

A PDMS chamber was prepared and covered with a PET film. Then the PDMS chamber was filled with the prepolymer through the inlet hole. Next, a photomask was placed on the PET substrate and exposed to UV light. The uncured prepolymer was washed with ethanol.

### SEM characterization

SEM images were obtained using an EM-30 scanning electron microscope (COXEM, Republic of Korea), with an acceleration voltage of 10 kV. A Fusion Cure 360 (MINUTA Tech, Republic of Korea) UV curing and patterning device (5.0 mW cm^−2^) was used to cure photocurable materials.

### UV-vis spectrophotometry measurements

Absorbance was measured using a Nano-MD PDA UV-Vis spectrophotometer (Scinco, Republic of Korea). Absorbances were measured in the wavelength range of UV light (300–500 nm), and molar extinction coefficients were calculated using the Beer–Lambert equation.

### Skin-penetration performance

Cadaver porcine skin was obtained from a local butcher shop, cut, and secured to bare PDMS using pins. The coating solution was prepared using a mixture of sulforhodamine B and 1 wt% of sodium dodecyl sulfate (SDS) for uniform coating of MNs. The MNs were coated with the solution by 10 min immersion, dried for 2 h, and inserted into the skin for 10 min. The residual dye was removed after penetration.

## Results and discussion

### Fabrication of cone-shaped MNs *via* free radical polymerization

**Fig. 1:**
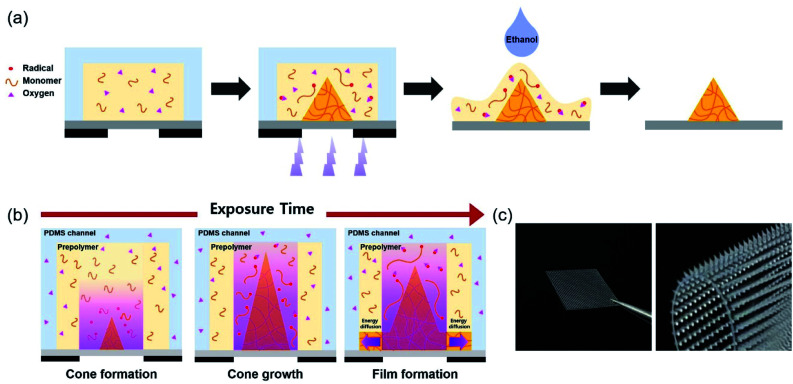
(a) Schematic illustration of the fabrication process of cone-shaped MNs using FRP. (b) Schematic illustration of formation of different MN shape asshapes with increasing exposure time increases. (c) Photographs of fabricated cone-shaped MNs, bonded on a flexible PET substrate.

Cone-shaped MNs were fabricated using photolithography, which is a simplified version of the conventional process using the principle of free radical polymerization (FRP). FRP is classified into three significant steps: initiation, propagation, and termination. In the initiation step, two radicals are generated from PI on exposure to UV light.^20^ Then, in the propagation step, the unstable radicals stimulate the functional groups of the prepolymers and are added to the unsaturated chains.^21^ Subsequently, termination occurs in two ways: integration of two chains, which leads to the elimination of radicals (coupling), or the formation of two chains from two dead chains by hydrogen abstraction (disproportionation), stopping polymer chain elongation. In this study, PEGDA prepolymer with different molecular weights was synthesized as a biocompatible material and PIs, with different absorption coefficients, followed by prepolymer and PI mixing. Fig. 1a shows a schematic representation of the MN array fabrication process. First, a mixture of a prepolymer and PI was filled inside a PDMS chamber and a thin PET film. The prepolymer within the PDMS chamber was solidified by photopolymerization on exposure to UV light through a photomask. After photopolymerization, the PDMS chamber was detached from the PET film bonded with the MNs, and the uncured prepolymer was washed with ethanol. The fabricated MNs were fully cured on exposure to UV light for over 2 h.

Oxygen inhibition should be considered while using FRP to fabricate MNs.^22^ Oxygen diffuses from the surroundings, hindering the reaction between radicals and prepolymers, by depriving them of radicals. This is the principle, by which cone-shaped MNs are fabricated through photopolymerization. The PDMS chamber, being an oxygen-permeable material, allows oxygen diffusion to the reaction sites, inducing a concentration gradient of oxygen. Additionally, an active inhibition reaction occurs at the reaction site, consuming oxygen, which contributes to the oxygen concentration gradient. Placing the reaction site between the oxygen-permeable (PDMS) and oxygen-impermeable (photomask) layers causes oxygen diffusion in both vertical and lateral directions. Thus, polymeric MNs form a cone shape from the central part of the oxygen-impermeable layer.^23^ UV light, refracted at the interface of the photocured and uncured PEGDA prepolymers, also leads to conical growth. As the cone angle becomes steeper, finally reaching a critical angle, the reflection remains only at the interface, and structural changes occur, as shown in Fig. 1b.^24^Fig. 1c shows a picture of cone-shaped MNs, fabricated on a flexible PET film, using photopolymerization.

### Effect of prepolymer molecular weight

**Fig. 2:**
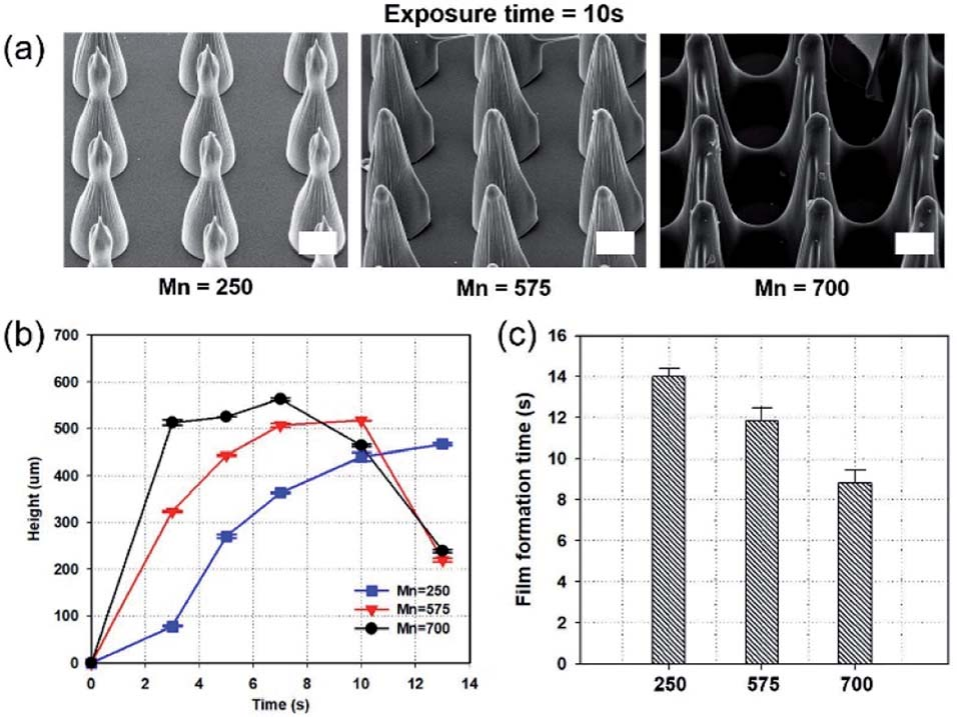
Fabrication of MNs using different molecular weights of PEGDA, with Darocur 1173, and 1 : 1 spacing ratio. (a) SEM images of fabricated MN arrays, using different molecular weights of PEGDA. (b) A graph of MN heights at gradually increasing exposure times. (c) A graph showing film formation time, by different molecular weights of PEGDA. Scale bar, 100 μm.

Fig. 2a shows scanning electron microscope (SEM) images of MNs fabricated by the photopolymerization of prepolymers, with different molecular weights, with the same exposure time (10 s). Darocur 1173 PI, and a photomask with holes possessing 150 μm diameter, and identical spacing as the diameter (spacing ratio = 1 : 1), were used. On fabricating MNs by the photopolymerization of PEGDA prepolymers, with a molecular weight of 700 (PEGDA 700), for 10 s, a film was formed between the MNs. In contrast, PEGDA with a smaller molecular weight (PEGDA 250) could form MNs without film formation. Fig. 2b shows MN heights using different exposure times and molecular weights. As the molecular weight increased, MN height also increased. The MNs fabricated with PEGDA 700 exhibited the fastest growth in the first 3 s, compared to the other MNs. Fig. 2c shows the incubation time for film formation with PEGDA of different molecular weights. The film formation times were 14, 12, and 9 s, for PEGDA molecular weights of 275, 575, and 700, respectively. Notably, among the three, the lowest molecular weight PEGDA exhibited the smallest polymerization rate, causing the slowest film formation.

The average degree of polymerization (*X*_*n*_) is calculated as follows:^25^1
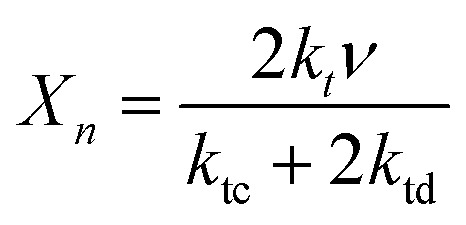
where *ν* is the kinetic chain length, which is the average number of monomer molecules added during polymerization, and *k* is the rate constant of termination (*k*_tc_ for coupling termination, and *k*_td_ for disproportionation termination). The molecular weight of the polymers was calculated as *M*_*n*_ = *M*_0_*X*_*n*_. According to this equation, the high molecular weight of the monomer increases the final molecular weight. Thus, high prepolymer molecular weight indicates a higher possibility of fast solidification, because dissolution rates are related to molecular weights.^26,27^ We note that gourd-shaped MNs are observed in Fig. 2a. Takahashi *et al.* reported the self-focusing effect in fabricating MNs by the reflection on the interface between the solidified and liquid prepolymer, and UV light rays diverged after reaching the critical point and converged again to form gourd-shaped MNs.^24^

### Effects of molar extinction coefficients of PIs

**Fig. 3:**
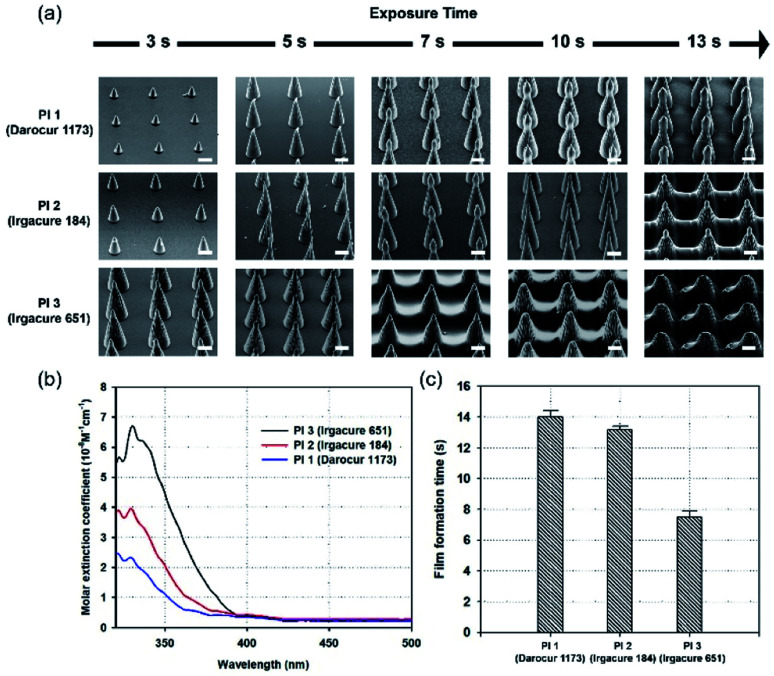
Fabrication of MNs with different PIs using PEGDA 250, and patterns. (a) SEM images of fabricated MNs, sorted by exposure time, and PIs. (b) A graph of PI molar extinction coefficient values in the 320–500 nm wavelength range. (c) A graph showing film formation time for PIs, using 1 : 1 spacing ratio of patterns. Scale bar, 100 μm.

PIs are very important for FRPs. A PI has a specific molar extinction coefficient value at a particular wavelength, which is one of the major factors affecting light absorption.^28^ Therefore, proper selection of PI is important to improve the photochemical reaction efficiency of photopolymerization.^29^Fig. 3a shows SEM images of MNs fabricated with three different PIs (Darocur 1173, Irgacure 184, Irgacure 651). The molecular weight of PEGDA (PEGDA 250) was kept constant to compare the effects of the PIs. The height of MNs formed using Darocur 1173 were the shortest, while those formed using Irgacure 651 were the longest, at the same exposure time. Additionally, films between MNs were easily formed when the PI was Irgacure 651. To investigate the effect of PIs on photopolymerization, the absorbances of the PIs in the wavelength range of UV light (300–500 nm) were measured. To calculate the molar extinction coefficient (*ε*) of each PI, the Beer–Lambert law was used:

2 *A* = *εlc*

where *l* is optical path length, and *c* is molar concentration.^30^ The molar extinction coefficient of Irgacure 651 was the highest, whereas the molar extinction coefficient of Darocur 1173 was the lowest. Thus, the MN growth rate during photopolymerization is proportional to the molar extinction coefficient of the PI used. Fig. 3c shows a graph of the film formation times of the MNs. Film formation times for MNs were proportional to the PI molar extinction coefficient values and were 14 s for those fabricated with Darocur 1173, and 13.2 s for Irgacure 184, and 7.5 s for Irgacure 651 (exhibiting the fastest film-formation).

### Effect of the spacing ratio between MNs

**Fig. 4:**
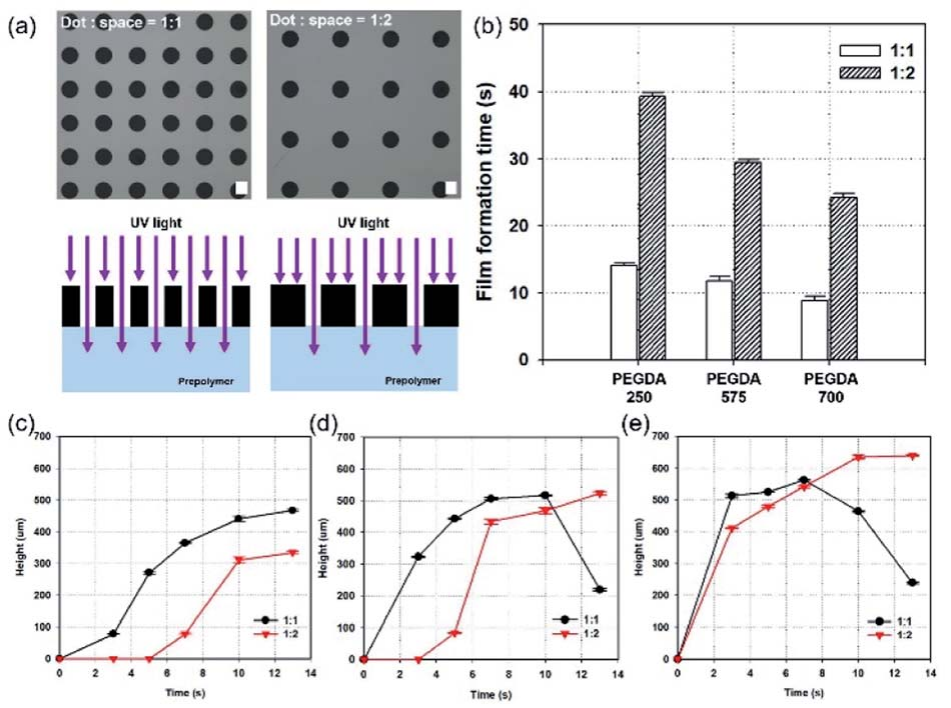
Fabrication of MNs with different spacing ratios of patterns. (a) Optical microscopic images of photomask patterns, and schematic illustrations of UV irradiation on the photomask, using 1 : 1 and 1 : 2 spacing ratios. (b) A graph showing film formation time by spacing ratios. Graphs showing MN heights, with 1 : 1, and 1 : 2 spacing-ratio patterns, using Darocur 1173, and (c) PEGDA 250, (d) PEGDA 575 and (e) PEGDA 700. Scale bar, 100 μm.

In addition to studying the effect of chemicals on photopolymerization, geometric factors in the design of photomasks were investigated. An MN array with two different spacing ratios (diameter-to-spacing) was fabricated to examine growth rate and film formation. Two different photomask patterns, with the same diameter (150 μm), but different spacing ratios (diameter : spacing) of 1 : 1 and 1 : 2, were used. Fig. 4a shows optical images of the photomask (black: hole, gray: space), and schematic images comparing the amount of UV irradiation for the spacing ratios of 1 : 1 and 1 : 2 (white: hole, black: space). Fig. 4b shows the film-formation time using patterns with different spacing ratios, sorted by the molecular weight of PEGDA. The patterns with a 1 : 1 spacing ratio transmitted a higher amount of UV irradiation than the patterns with a 1 : 2 spacing ratio. Hole arrays with higher densities enable greater light transmittance through the photomask, promoting higher interference, causing scattering and diffraction. This causes faster energy diffusion to the surroundings, leading to a higher growth rate, and faster film formation. Fig. 4c–e show the heights of MNs fabricated with Darocur 1173, and PEGDA with molecular weights of 250, 575, and 700, respectively. Patterns with a high spacing ratio showed faster MN height growth and film formation compared to patterns with a low spacing ratio (Fig. S1†). It is noted that the maximum height of the MNs is ∼640 μm, fabricated with PEGDA (Mn: 700), Darocur 1173, and a spacing ratio of 1 : 2.

### Penetration test using cadaver porcine skin

**Fig. 5:**
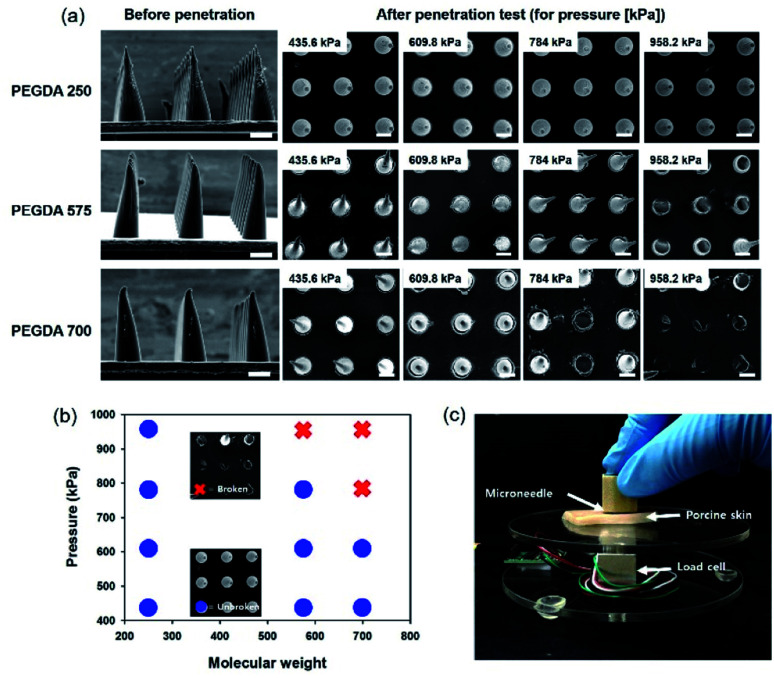
(a) SEM images of MNs before and after the penetration test on the porcine skin according to the PEGDA molecular weight and shear pressure. (b) A graph showing the condition to be stable or broken after the penetration test according to the PEGDA molecular weight and shear pressure. (c) An experimental setup to measure the applied force to MNs using a load cell. Scale bars represent 100 μm.

To study the mechanical stability of MNs with different PEGDA molecular weights, we conducted a penetration test with MNs of the same height. As shown in Fig. 5a, MNs with different molecular weights and heights (340 μm) were prepared by controlling the exposure time. Subsequently, we pressed a porcine skin, placed on a load cell with an MN film, to measure the applied pressure. To compare the mechanical stability, we applied the shear pressure by pushing it in the lateral direction after contacting MNs on the porcine skin. As shown in Fig. 5a, MNs with PEGDA 250 were not broken until we applied 958.2 kPa, however, MNs with PEGDA 575 and PEGDA 700 were broken in the test. Fig. 5b shows a graph of the stability of MNs with different molecular weights, and Fig. 5c shows the experimental setup used to measure the applied pressure. In a previous study, PEGDA 700 exhibited a higher value of elongation at break than that of PEGDA 250 under the same UV exposure time.^31^ In the current study, the UV exposure time was different for fabricating MNs with the same height. The short exposure time of PEGDA 700 for fabricating MNs without film formation induced a mechanical weakness because polymerization was not enough, although we conducted the additional UV exposure after rinsing to remove the uncured prepolymer.

**Fig. 6:**
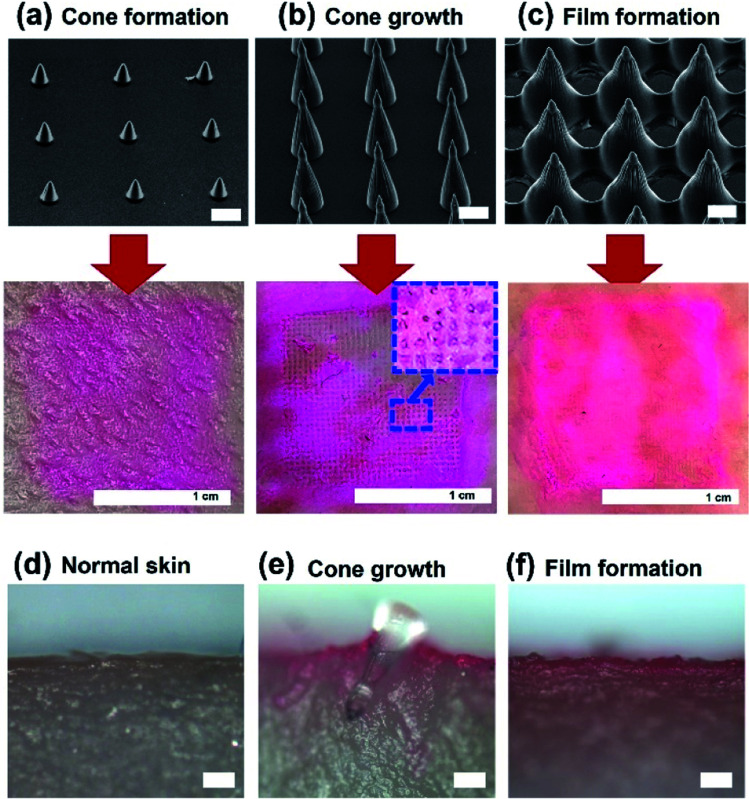
Penetration test results of MNs on the porcine skin. Inserted MNs were fabricated with PEGDA 250, Darocur 1173, and 1 : 1 spacing-ratio patterns. (a) 78 μm, (b) 350 μm, and (c) 200 μm with film formation. Scale bars in SEM images represent 100 μm. The cone growth sample can penetrate the porcine skin. Cross-sectional images of porcine skin (d) before penetration, (e) after penetration with a cone-shape MN, (f) after penetration with an MN in the film formation condition. Scale bars in optical images represent 100 μm.

To investigate the penetration of high-aspect-ratio MNs, we examined the porcine skin after the penetration. A coating solution, prepared by mixing a dye (sulforhodamine B) and a surfactant (1 wt% of SDS), was used to confirm penetration. MNs were coated with the prepared solution using an immersion-coating method for 10 min and dried in air for 2 h. Fat and oil from the porcine skin were removed before penetration. MNs were inserted into porcine skin under 430 kPa for 10 min, and the residual dye was removed after penetration. Fig. 6a–c show SEM images of the used MNs, and pictures showing the cadaver porcine skin morphology after penetration. Three MNs, fabricated with PEGDA 250 and patterns with 1 : 1 spacing ratio, but different shapes (cone formation, cone growth, and film formation), and heights (78, 350, and 200 μm with film formation), were used. MNs with large heights exhibited better performance in the penetration test. However, on using MNs with film formation or with small heights, MNs did not penetrate the porcine skins. Fig. 6d shows the cross-sectional image of porcine skin before the penetration. After the penetration, we cut the skin penetrated by MNs by a razor before the detachment of MNs from the skin. Fig. 6e shows that an MN can penetrate the porcine skin. When we used MNs with film formation, the MN is detached during cutting by a razor, and Fig. 6f shows that the MNs did not penetrate the skin.

## Conclusions

In this study, conditions for the fabrication of high-aspect-ratio MNs, using photopolymerization with a photomask, were investigated. The effects of prepolymer molecular weight, PI absorption coefficients, and pattern spacing ratios on high-aspect-ratio MN fabrication were analyzed. High prepolymer molecular weights and PI molar extinction coefficients caused rapid height growth and film formation. Two different patterns with the same diameter (150 μm), and different spacing ratios (1 : 1 and 1 : 2), were used to study the geometric effects. Dense pattern arrays caused faster energy diffusion to the surroundings, owing to light scattering and diffraction. In the aspect of polymerization rate, PEGDA 700, patterns with a 1 : 1 spacing ratio, and Irgacure 651, are recommended for use. However, because penetrability is vital, PEGDA 700, with high fragility, is not suitable for use. A low molecular weight prepolymer (PEGDA 250) was used, for better robustness, even after the penetration test. High molar extinction coefficients induced a high MN growth rate, and short incubation time for film formation. Therefore, PEGDA 250 with high mechanical stability, and Darocur 1173 with high MN-formation controllability without film formation, and dense hole arrays, enabling higher growth rate, were optimum for penetration and hence MN fabrication. This work could guide prepolymer and PI selection, and enable tuning of the geometric factors of a photomask, to facilitate the use of new biocompatible materials to fabricate high-aspect-ratio MNs, and to design photomasks.

## Conflicts of interest

There are no conflicts to declare.

## Supplementary Material

RA-012-D2RA00189F-s001
