# Supplementary material for: Investigation on photopolymerization of PEGDA to fabricate high-aspect-ratio microneedles
Source: RSC Adv. 2022 Mar 28;12(16):9550–5. doi: 10.1039/d2ra00189f (PMC8985357; doi:10.1039/d2ra00189f)
Supplement: RA-012-D2RA00189F-s001 [file RA-012-D2RA00189F-s001.pdf]

Electronic Supplementary Material (ESI) for RSC Advances.
This journal is © The Royal Society of Chemistry 2022

Supplementary Information

Investigation on Photopolymerization of PEGDA to Fabricate High-Aspect-Ratio Microneedles

Sohyun Kim,[a†] Hyemin Lee, [b†] Hyewon Choi,[b] Kee-Youn Yoo[*b] and Hyunsik Yoon[*ab]

[a.]Department of Nano Bio Engineering, Seoul National University of Science and Technology, Seoul, 01811, Republic of Korea

[b.]Department of Chemical & Biomolecular Engineering, Seoul National University of Science and Technology, Seoul, 01811, Republic of Korea Email: hsyoon@seoultech.ac.kr and kyyoo@seoultech.ac.kr

† These authors contributed equally to this work.

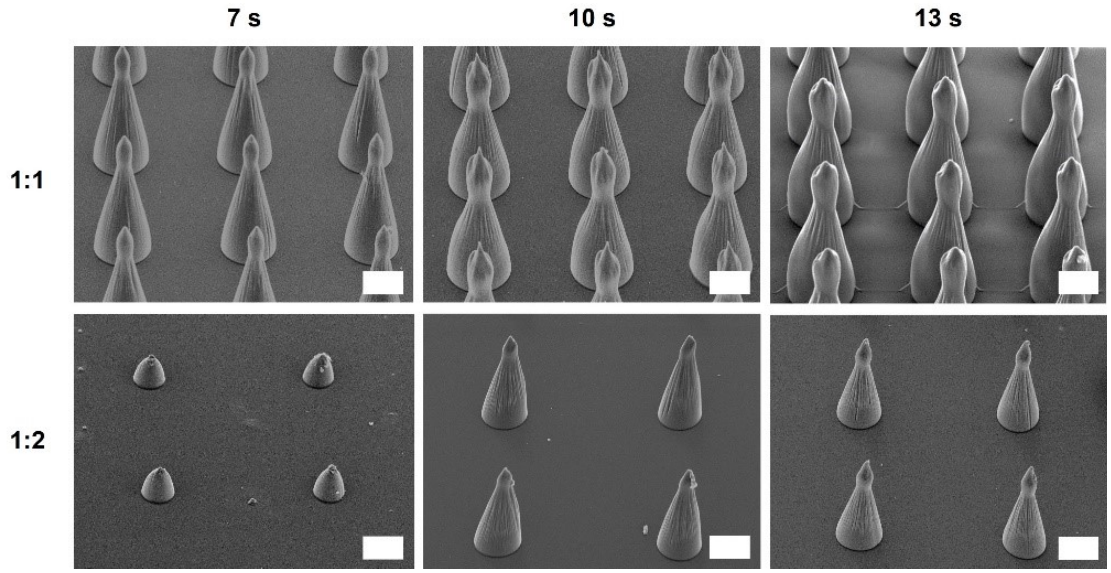

Figure S1. SEM images of MNs according to the spacing ratio between MNs and UV exposure time. Scale bar = 100 µm.
